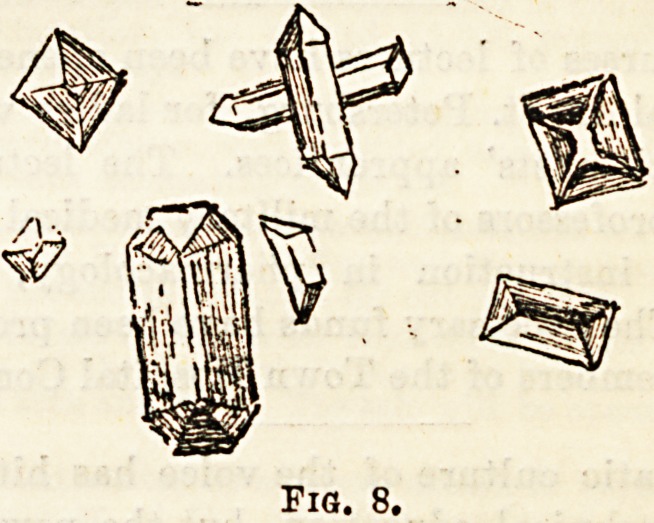# The Microscope in Medicine

**Published:** 1891-04-25

**Authors:** Frank J. Wethered


### THE MICROSCOPE IN MEDICINE.

By Frank J. Wethered, M.D.

### XII.—THE EXAMINATION OF URINARY DEPOSITS.

*(Continued.)*

*Oxalate of Lime* (Fig. 7). — The crystals of this substance are transparent, colourless, strongly reflecting octahedra; owing to their shape they are known as "envelope

Fig. 7.

crystals." Occasionally other forms are seen designated the "dumb-bell" form. They are of no great pathological importance, and are often seen in large numbers after a vegetable diet.

*Leucin.*—This substance when seen in urinary deposits consists of yellowish oily-looking drops. In order to demonstrate their nature they should be dissolved in alcohol, where pure leucin crystallises out into shining delicate plates.

*Tyrosin.*—This body nearly always occurs together with the last-named. Its crystals are, however, more characteristic, taking the form of long slender needles, arranged in a radiating manner. Leucin and Tyrosin are found in certain affections of the liver, being especially charateristic of acute yellow atrophy. It is also found in leucocythaemia and acute phosphorus poisoning.

*Cystine.*—This is a rare deposit. Its only importance is its association with calculi of the same substance. These crystals take the form of six-sided plates, very often adhering to one another. They may easily be mistaken for uric acid crystals, but may be distinguished from them by being soluble in ammonia, and insoluble in acetic acid.

*Sulphate of Lime.*—These crystals are also rarely seen. In shape they are long, slender prisms, with oblique ends, and lie together in the form of bundles.

*Urates.*—These usually form an amorphous deposit, but the urate of soda is occasionally thrown down in the "hedgehog" crystals. Their occurrence is of no pathological interest, being often met with in the urine of healthy persons.

Sediments from Alkaline Urine.

*Triple Phosphates* (Fig. 8).—These take the form of "coffin-lid"-like crystals of various sizes, their shape being

Fig. 8.

modified in various ways, according to the alkalinity of the urine.

*Neutral Phosphate of Lime.*—This substance appears as peculiar wedge-shaped crystals, gathered together in star-like groups, and are commonly known as "Stellar" phosphates.

*Basic Magnesium Phosphate.*—This body does not often crystallise, but when thus found takes the shape of elongated rhombic plates, with rounded ends.

The various phosphates occur under such varying circumstances that their presence is of little aid in diagnosis.

*Urate of Ammonia.*—Crystals of this substance are very characteristic, assuming the form of dark spheres beset with spicules. When acetic acid is added they dissolve, free uric acid afterwards crystallising out.

## Figures and Tables

**Fig. 7. f1:**
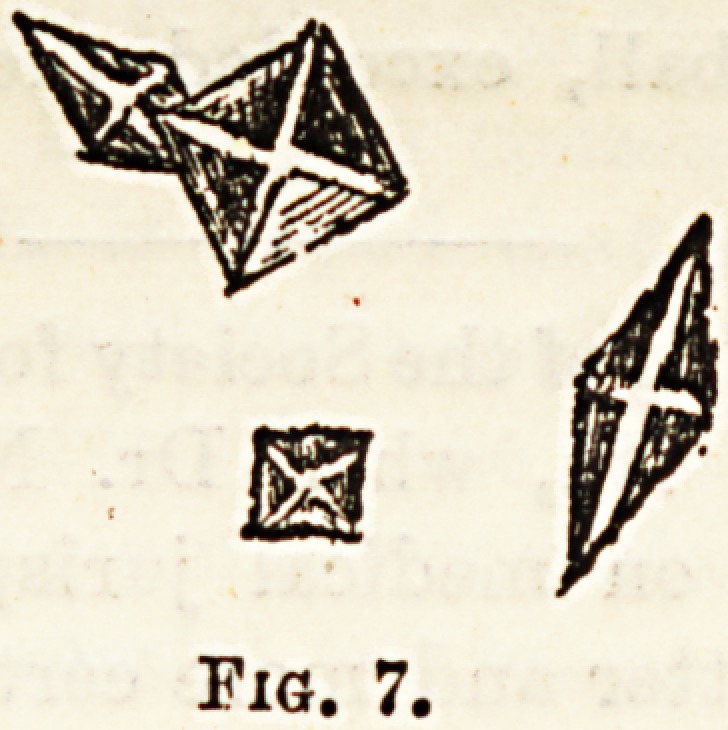


**Fig. 8. f2:**